# Measuring the state of aquatic environments using eDNA—upscaling spatial resolution of biotic indices

**DOI:** 10.1098/rstb.2023.0121

**Published:** 2024-06-24

**Authors:** Rosetta C. Blackman, Luca Carraro, François Keck, Florian Altermatt

**Affiliations:** ^1^ Department of Evolutionary Biology and Environmental Studies, University of Zurich, Winterthurerstr. 190, Zürich 8057, Switzerland; ^2^ Department of Aquatic Ecology, Eawag, Swiss Federal Institute of Aquatic Science and Technology, Überlandstrasse 133, Dübendorf 8600, Switzerland

**Keywords:** biomonitoring, eDNA, hydrological model, macroinvertebrates

## Abstract

Aquatic macroinvertebrates, including many aquatic insect orders, are a diverse and ecologically relevant organismal group yet they are strongly affected by anthropogenic activities. As many of these taxa are highly sensitive to environmental change, they offer a particularly good early warning system for human-induced change, thus leading to their intense monitoring. In aquatic ecosystems there is a plethora of biotic monitoring or biomonitoring approaches, with more than 300 assessment methods reported for freshwater taxa alone. Ultimately, monitoring of aquatic macroinvertebrates is used to calculate ecological indices describing the state of aquatic systems. Many of the methods and indices used are not only hard to compare, but especially difficult to scale in time and space. Novel DNA-based approaches to measure the state and change of aquatic environments now offer unprecedented opportunities, also for possible integration towards commonly applicable indices. Here, we first give a perspective on DNA-based approaches in the monitoring of aquatic organisms, with a focus on aquatic insects, and how to move beyond traditional point-based biotic indices. Second, we demonstrate a proof-of-concept for spatially upscaling ecological indices based on environmental DNA, demonstrating how integration of these novel molecular approaches with hydrological models allows an accurate evaluation at the catchment scale.

This article is part of the theme issue ‘Towards a toolkit for global insect biodiversity monitoring’.

## 1. Introduction

**Figure 1.**
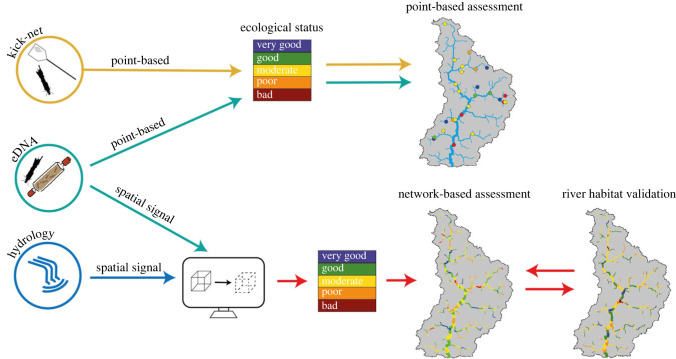
Conceptual diagram of how point-based kick-net assessment of macroinvertebrates gives only very localized assessments of ecological river status. In contrast, application of environmental DNA (eDNA) and a hydrological transport model allows for a space-filling projection of species' possible occurrence, and subsequent calculation of ecological status. Traditional kick-net sampling (followed by microscopy analysis) is the established method used for assessing the ecological status of a freshwater ecosystem (e.g. a river network), relying on the collection of scoring taxa to calculate biotic indices. These point samples can be used to determine the ecological status of a river stretch as a categorical indicator (i.e. very good, good, moderate, poor or bad) based on prior knowledge of species tolerance or comparing the community assemblage (observed) with the community expected under pristine condition. Here, we demonstrate how molecular samples (e.g. eDNA) can either be used for a point-based assessment (similar to current methods) or combined with hydrological information and the eDNA Integrating Transport and Hydrology (eDITH) model to upscale the ecological assessment of the river from points to the whole network.

Often the state of an ecosystem is not assessed by the entirety of its biodiversity but by a series of bioindicator taxa (figure 1). These bioindicator groups are well known to react to ecosystem stressors, such as organic pollution, nutrient application and habitat modification via their presence, abundance or lack thereof [1]. Macroinvertebrates (those invertebrates visible to the naked eye) are considered key indicators of the state of freshwater environments. Their presence or absence in a focal water course, along with that of other bioindicator groups, such as fish or diatoms, is transformed into environmental indices to assess the ‘health’ or status of the ecosystem [1,2]. For example, macroinvertebrates can be used to identify anthropogenic pressures, such as fine sediment loading (via the Proportion of Sediment-sensitive Invertebrates index, PSI; [3]), nutrient enrichment (Whalley Hawkes Paisley Trigg index, WHPT; [4]), drought (via the Lotic Invertebrate index for Flow Evaluation index, LIFE; [5]) or even the integrated effects of hydrodynamics, chemical/toxic effects (e.g. Index Biologique Suisse, IBCH index; [6]), leading to routine detection and monitoring of anthropogenic pressures in freshwater ecosystems.

These biotic indices, alongside many others, form the foundations of several legislative and directive reporting methods, commonly referred to as biomonitoring. However, the methods of sample collection and index calculation can vary from country to country or even from region to region [7,8]. For example, in Germany, freshwater macroinvertebrates are monitored under the PERLODES protocol [9], whereby a kick-net sample is collected and a fixed number of individuals are identified (e.g. 360 specimens) to species level. However, in England all macroinvertebrate specimens collected in a 3 minute kick-net sample are sorted to family level [10]. Similarly, calculations of their respective ecological assessment differ. In Germany a multimeric approach is used, combining taxonomic richness, species sensitivity and community assemblages with predicted community composition. This compound metric is then used to assess the status of the water body [8]. Instead, in England multivariate analysis is combined with a biotic index (WHPT) to compare the expected community composition of the site in pristine condition, based on the physical parameters of the water body at that site, e.g. altitude, distance from source, flow rate, substrate type, etc. [11]. Additionally, in England the Average Score Per Taxa (WHPT–ASPT) is then derived from not only the sensitivity of the taxa identified, but also from their abundance, whereby abundances are separated into the following classes: AB1 = 1–9 individuals, AB2 = 10–99 individuals, AB3 = 100–999 individuals and AB4 ≥ 1000 individuals [4]. Alternatively, in Switzerland the IBCH index is used for biomonitoring of macroinvertebrates where the ecological assessment is derived exclusively from family-level sensitivity scores and does not require further modelling of expected community compositions. With more than 300 known bioindicator methods used under the Water Framework Directive (WFD) alone [7], these methods are not only costly in terms of time and financial outlay, but the disparity in biomonitoring approaches could lead to differing results [12,13].

Furthermore, biomonitoring focusses on sampling individual points within an ecosystem. Yet river systems are inherently spatially structured as dendritic networks [14,15]. By current approaches, they are described solely on a selected number of sites, with large stretches between these points not covered and with any influence from the surrounding or upstream habitat being disregarded. This is counterintuitive to the assessment of an ecosystem characterized by diverse and changing habitats characteristic of a riverine network. It is therefore appropriate to consider alternative methods to assess the state of riverine systems, which could rectify some of the current constraints.

## 2. The advancement of molecular tools

Within the past decade, the use of molecular tools has taken the biodiversity research world by storm. Often referred to as a ‘game-changer’ [16], the ‘efficiency and sensitivity’ [17] of molecular tools have changed the possible ways we explore and describe biodiversity and whole ecosystems. For these methods, DNA is extracted from either a community bulk sample (e.g. a kick-net sample) or from environmental samples (e.g. water), the latter being known as environmental DNA (eDNA; see [18,19]). Importantly, the use of eDNA for assessing biodiversity of macroinvertebrates largely relies on fragments of DNA shed by organisms into an ecosystem, thus requiring no specimens to be collected or killed in its application. This consideration renders the use of eDNA distinctly favourable for conservation of threatened freshwater biodiversity. In freshwater ecosystems there has been a notable growth in the number of eDNA studies since its first applications [20], with more than 400 peer-reviewed articles published on aquatic macro-organism biomonitoring in the past decade [17]. These applications are divided into two possible methods for biomonitoring: first, the use of eDNA for single species detection, where a target organism is the subject of a highly sensitive assay; second, community analysis, which uses a more generic broad approach to detect taxa within a certain taxonomic grouping. Single species detection is the most widely published application for the use of eDNA [17], with a substantial number of species-specific primers being developed and validated for rare, cryptic, elusive or invasive alien species [17,21]. Although this form of molecular biomonitoring could be of great benefit, particularly in the detection of invasive alien species at the beginning of an invasion [22,23], uptake in terms of biomonitoring with single species eDNA assays has been lagging woefully behind the developments of this method. The most advanced use is arguably the monitoring of the protected great crested newt (*Triturus cristatus*, Laurenti, 1768) in the UK [24], but as yet no macroinvertebrates are monitored using eDNA for regulatory use. However, in terms of aquatic environment assessment, metabarcoding holds significant promise for biomonitoring. Here, technology advancements in sequencing platforms known as Next Generation Sequencing (NGS) have greatly improved the workflow [25] and offer the capacity to generate large datasets focused on the detection of whole groups, such as macroinvertebrates, diatoms or fish. However, despite this development, the transition into stakeholder applications and governmental monitoring to assess the state of the aquatic ecosystems is lagging behind [25,26].

## 3. Hurdles for ecological assessment with DNA-based tools

Despite the large improvements in understanding eDNA metabarcoding in recent years, the fact that these advancements have not leaped from research to the application of DNA-based monitoring methods could be attributed to the explosion in method development. Rapid methodological advances have brought many possible avenues of application, but also lack of standardisation across the field [26–28]. Much of the early eDNA method development focused on the ‘renovation’ of conventional bioindicator groups for molecular-based approaches (e.g. ‘like for like’ replacements [25]). This led to a significant amount of comparative work between traditional and molecular tools [29], which showed overall congruence between methods, yet with some notable differences. Specifically, this congruence was relatively good for some bioindicator groups (in particular fish and diatoms), yet more variable for others (e.g. macroinvertebrates). The variability in detection of macroinvertebrates is essentially due to using a single primer to detect a polyphyletic group of taxa that are grouped together under the umbrella term 'macroinvertebrate' (e.g. Arthropoda, Annelida, Porifera, Cnidaria, Mollusca and Bivalvia; [30]) and are considered a single bioindicator group because they are all recovered by the same kick-net sampling method [27]. Therefore, nuanced approaches and a break from traditional groupings will likely revolutionize this form of biomonitoring [25].

A further consideration of DNA-based approaches for biomonitoring is the accessibility and taxonomic identification of sequences [31,32]. Compared to current methods, the importance of an accurate reference database can be seen as akin to the state of a sampling net: if the net has holes in it, specimens will be lost during sampling; equally, if the database is not complete, species' sequences cannot be assigned and thus are lost from the analysis ([33]; but see [32]). For those taxa that are currently used in biomonitoring programmes, fish are again a group that is particularly advanced in terms of comprehensive sequence reference databases [33]. However, this coverage is far from globally consistent [31]. For other taxonomic groups, progress is ongoing and improvements are often a direct result of eDNA sampling campaigns. Although these developmental hurdles pose issues, there has been and continues to be huge development via national and international consortia (e.g. UK DNA Working Groups and DNAqua-net). Contributions by these consortia have gone a long way in the research and standardization of many methodological aspects [34], in parallel to specific targeted studies to improve the methods and interpretation of DNA-based tools specifically for macroinvertebrate biomonitoring [27].

## 4. Opportunities for ecological assessment with eDNA-based tools

Bioindicator groups were originally selected for ecological assessments according to their ease of collection and pre-existing ecological knowledge of species. However, the use of eDNA sampling would allow sample collection to go beyond what is currently achievable in terms of repeated biomonitoring. NGS technologies have revolutionized potential biomonitoring campaigns by enabling the simultaneous sequencing of a large number of samples, gaining unprecedented amounts of data from samples collected at both large spatial and temporal scales [35], while being cost-effective. This is an advantage particularly for river networks and large or stratified lakes, which can now be sampled in a spatially replicated manner, and allows the inclusion of sampling conducted across seasons. Both aspects are particularly important when studying groups like macroinvertebrates, with strong seasonal variation [36–38].

Environmental DNA-based biomonitoring is also not restricted to traditional bioindicator groups. By departing from conventional sampling, it allows for the simultaneous inclusion of not only the multiple bioindicators (via resequencing of the same eDNA samples for different groups; see [37]), but also novel bioindicators like microbes [39] or even groups that have been overlooked by traditional taxonomic constraints [40]. Additionally, taxonomy-free approaches offer an opportunity to utilize molecular data beyond the constraints of known taxa. This could overcome the constraints of reference database completion and allow molecular units to be calibrated with known or emerging environmental stressors [25,41]. The wealth of nuanced information obtained through DNA-based methods, combined with machine learning techniques [30,42], could also introduce new tools for characterizing environments in the face of emerging pressures—particularly at a time of global climate change and shift of communities. Therefore, a break from traditional bioindicator groupings is the largest opportunity for policy and end-users to consider. While this must be done in a standardized manner, with parallel sampling programs (traditional and molecular) and intercalibration to prevent multiple versions of the same tool being developed (e.g. WFD monitoring methods), such work will likely revolutionize biomonitoring [25].

In the context of biomonitoring, the potential for application of eDNA relies on what could be considered its ‘black box’, i.e. what spatial extent the eDNA signal represents [43]. As eDNA sampling is not reliant on the collection of a specimen, the genetic material collected is subject to hydrological transport by the water current, as well as various abiotic factors such as temperature and UV exposure that impact its decay [43–45]. Consequently, the duration of the signal detectability and the distance travelled by DNA within this time require nuanced methods for interpretation [46–50]—specifically, the integration of hydrological information into taxon detection. However, considering the spatial extent of the eDNA signal should be seen as a huge advantage. By using suitable network modelling approaches that explicitly consider transport of eDNA (e.g. eDITH; [51–53]), both the point-based (the current method used for biomonitoring) and the spatial projection of the ecological status across a whole river catchment (i.e. the state of a whole ecosystem) can be determined. This capacity provides enhanced understanding of not only the threats to biodiversity within specific areas of the river network, but also how these factors may affect the community at a regional level from upstream to downstream. Ultimately, ecological assessment of an ecosystem must rely on fast and effective methods of describing the biodiversity present. In this respect, the use of eDNA data for biomonitoring not only satisfies these requirements, but it also allows us—when interpreted via hydrological models—to project pointwise eDNA samples spatially beyond their collection point and reflect biodiversity as well as possible indicators derived thereof at the ecosystem level. Traditional ecological status assessments have hitherto been spatially restricted to the sites where the bioindicator group—here aquatic macroinvertebrates—have been sampled. Using eDNA from aquatic macroinvertebrates, we can spatially project the assessment to the network level and fill the spaces between point samples with ecological status predictions (figure 1).

Here, we provide a proof-of-concept of how the combination of eDNA and hydrological modelling allows predictions of the ecological state of a river at the whole river network scale, with a resolution of a few hundred-metre reach lengths. Specifically, we demonstrate the use of eDNA data in combination with the eDITH model [51–53]. We explicitly determine the ecological status of a complete river network, based on the occurrence of macroinvertebrate DNA taken from water samples. We then ground truth the predicted ecological status across a whole catchment with river habitat information, thus demonstrating the reliability of this approach.

## 5. Case study

The Thur River catchment (740 km^2^) is a pre-alpine river in North-West Switzerland. The catchment is routinely monitored under the Swiss-wide river monitoring campaign (Nationale Beobachtung Obergewässerqualität (NAWA) by BAFU), including macroinvertebrate and river habitat. As part of these traditional biomonitoring schemes, the biological status is resolved using the IBCH index from macroinvertebrate samples [6]. Overall, this method includes 142 taxonomic units of varying taxonomic resolution that are considered across Switzerland, mostly composed by families. Within any given river catchment often only a fraction of all taxonomic units is detected, which still enables the calculation of ecological indices. The index is based largely on species' tolerance scores to abiotic stressors (pollution, habitat modification). Taxa (typically identified to family level) are grouped into Group Indicators (GI) ranging from 1–9 based on their ecological value, with GI = 9 being the most ‘valuable’ or sensitive group (e.g. this includes sensitive taxa such as Chloroperlidae, Perlidae, Perlodidae or Taeniopterygidae, which, if present, results in the value of 9 being attributed to the site). This system is reliant on a minimum number of individuals in the sample as opposed to an abundance weighting (i.e. 3 individuals; [6]). Furthermore, the presence of sensitive species is modulated by the total diversity present at a site, which ultimately allows one to calculate the IBCH index. As a weighted system, the IBCH index is generated based on values assigned to the sensitivity as well as the diversity of key macroinvertebrate families. Details on the calculation of the IBCH index are reported in the electronic supplementary material, Information.

To demonstrate the assessment of eDNA-based ecological status estimates at the catchment scale, we compared kick-net-derived IBCH ecological status with those generated from eDNA, and we subsequently validated our predictions with river habitat surveys carried out over the entire Thur catchment. To do this, we first considered 61 eDNA samples as collected by Mächler and colleagues [54]. In brief, these samples were amplified for macroinvertebrate DNA using the *COI* barcoding region specified by [55] and [56] primers for invertebrates. The samples were then sequenced on an Illumina MiSeq (see electronic supplementary material, Information and [54] for full details). In our study we reran the taxonomic assignment (see electronic supplementary material, Information) of the reads generated by Mächler *et al.* [54] and carried out analysis only on reads from macroinvertebrate taxa included in the IBCH index [6]. Second, for each of the taxonomic units considered by the IBCH index and found in the eDNA dataset, we applied the eDITH model to the Thur catchment network discretized into 1839 reaches (see details in the electronic supplementary material, Information) and thus derived predicted maps of detection probability within these reaches. We finally transformed these into predicted presence/absence maps by applying a threshold of 0.5 (see electronic supplementary material, figure S1 for a sensitivity analysis on this value).

To assess our predictions, we first compared the eDNA-based IBCH index with the independently observed historical kick-net-based IBCH index derived from 25 sites sampled by the environmental agency of Canton St. Gallen between 2016 and 2020. Second, to validate the eDNA-based IBCH predictions, we used data on the watercourse structure (river habitat status) based on visual surveys of the river reaches carried out by the cantonal and federal environmental agencies in Switzerland. This dataset included 4472 individual river reaches within the Thur catchment that were visually inspected and assigned to a status category based on ecologically important features, such as the bank structure or substrate type (for full methods see electronic supplementary material).

**Figure 2.**
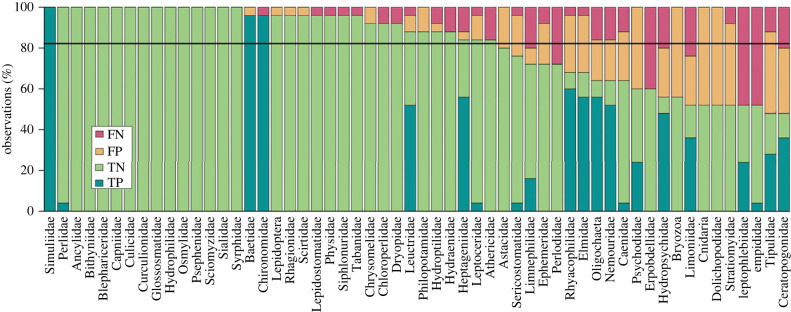
Comparison of the model-based presence/absence predictions for the 56 taxa identified in the eDNA dataset against measured kick-net-based presence/absence data. For all taxa found at the 25 sites (Points in figure 2*b*) the congruence between the two methods is shown in terms of true positives (TP; i.e. the presence predicted by the model matches the occurrence in the kick-net dataset), false positives (FP; i.e. the model predicts presence but the taxon was not found by kick-net), true negatives (TN) and false negatives (FN). Importantly both methods have an inherent sampling error, thus contribute comparably to false negatives and false positives. The horizontal black line shows the average accuracy across the 56 taxa, which is equal to 81.9%.

**Figure 3.**
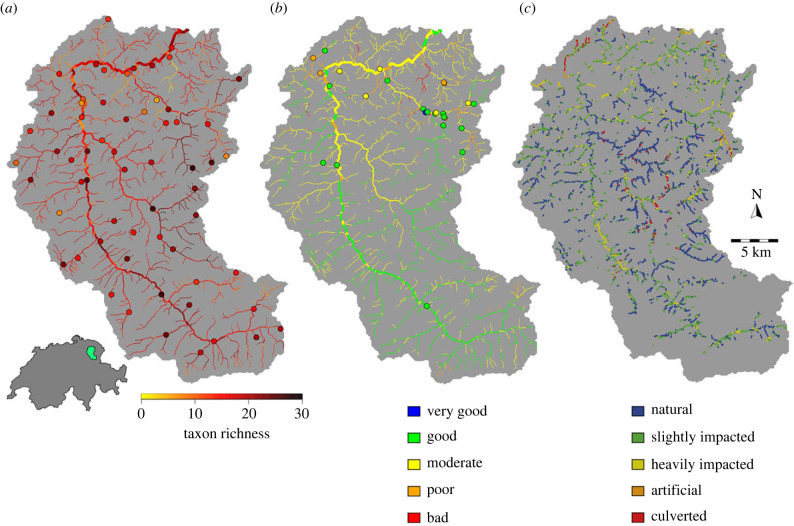
Spatial maps of biodiversity and ecological status in the Thur. (*a*) Richness of taxa used in the estimation of the IBCH index (filled dots: eDNA data; river reaches: eDITH model predictions). The inset shows the location of the Thur catchment within Switzerland. (*b*) IBCH index values based on the modelled taxon distribution (river reaches) and measured by the Swiss Federal Office for the Environment (filled dots). (*c*) River habitat status as derived from visual surveys of the habitat status of individual reaches (see Methods and SI).

**Figure 4.**
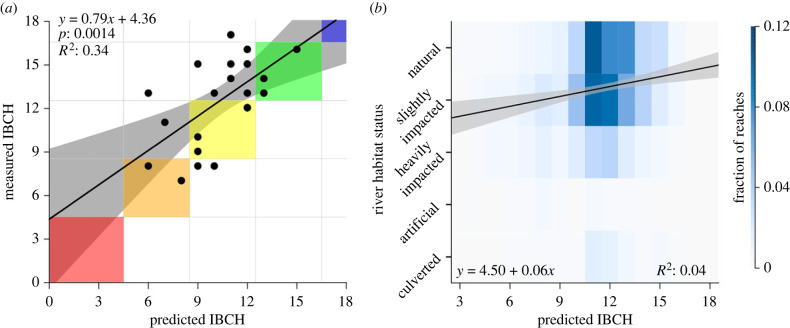
Regressions of indices derived from empirical measurements on modelled ecological indices and validation of the eDITH model. (*a*) Relation between measured kick-net-based IBCH and predicted eDNA-based IBCH values at the corresponding reaches (figure 3*b*). Linear regression lines are shown in black. The grid and shaded boxes along the diagonal identify the IBCH levels corresponding to the colours of figure 3*b*. (*b*) Two-dimensional histogram of paired values of the predicted IBCH index (figure 3*b*) for the 4472 river reaches and the identified river habitat status (figure 3*c*; see electronic supplementary material). Each rectangle shows the fraction of reaches with a given river habitat status and IBCH index [1–20] value. The 95% confidence band was fitted to the 4472 points underlying the two-dimensional histogram. (See electronic supplementary material for further details.)

Overall, the metabarcoding dataset contained 56 out of the 142 macroinvertebrate taxonomic units used for the calculation of the IBCH index, while the IBCH kick-net data from the same study region contained 67 of these 142 taxa, with an overlap of 40 taxa between the two datasets. For the 56 taxonomic units found by metabarcoding, we found an average accuracy (i.e. sum of fraction of true positive and true negative predictions) between eDITH-based and kick-net-based presence/absence of 81.9% (see figure 2 for a taxon-by-taxon breakdown). The measured IBCH (kick-net-based) at the 25 sites ranged from 7 to 16, whereas the eDNA-based richness of IBCH-relevant taxa observed at the 61 sites ranged from 6 to 27. The richness predicted by the eDITH model at the 1839 reaches ranged from 0 to 30 (figure 3*a*), with the predicted IBCH index at the corresponding kick-net sites ranging from 0 to 17 (figure 3*b*). A linear regression between the measured and predicted IBCH at those sites showed a significant fit (*R*^2^ = 0.34, *F*_1,23_ = 13.3, *p* = 0.001; figure 4*a*), with a fitted slope of 0.79 (with 95% confidence interval of ± 0.43) not statistically distinct from the expected value of 1. Furthermore, the fitted intercept 4.36 ± 4.57 did not depart significantly from the expected value of 0. This shows the link between predicted and measured IBCH, with increases in measured IBCH matched by comparable increases in predicted IBCH (hence the slope close to 1). However, our predictions tend to underestimate the measured IBCH (figure 4). The disparity between the two biotic indices is in part due to the lack of sites with low measured IBCH value (hence the confidence interval for the fitted intercept overlapping 0 only by a small margin), and in part to methodological constraints (as discussed under *Hurdles for ecological assessment with DNA-based tools* section). Specifically, we observed a mismatch in the detection of some ecologically relevant taxa (with GI > 6) that were largely found by kick-net but not by eDNA, such as Taeniopterygidae (GI = 9, found in 15 sites) and Polycentropidae (GI = 7, found in 16 sites), while no ecologically relevant taxa in proximity to the IBCH sampling sites were exclusively found by eDNA.

We further validated our predictions of the ecological status of individual river reaches (figure 3*b*, lines) by using the river habitat classifications carried out in the Thur catchment (figure 3*c*). We found that higher eDNA-based IBCH index correlated with less impacted classifications of river habitat (*R*^2^ = 0.04, figure 4*b*). Although this association is not strong, it is important to recognize that the habitat status serves only as an indirect measure of the ecological status, and consequently it is reasonable to expect the correlation to be low. Further methods to validate the findings of the model would require extensive field sample collection of macroinvertebrates (along the entire river length), which is unfeasible. However, this proof-of-concept takes considerable steps towards using eDNA and hydrological models to produce relevant and usable ecological assessment of an entire river network, thus providing end users with detailed information beyond current point-source methods.

## 6. Outlook

In the context of conservation and protection of ecosystems, the primary goal is to bend the curve of biodiversity decline [57]. This is particularly relevant for freshwater systems, which are highly susceptible to anthropogenic pressures and have failed to recover despite international efforts [58]. To do this, fast, effective and accurate data must be used for the identification and mitigation of ecosystem threats, and the prevention of further declines in biodiversity and the associated ecological status [17,35,59]. Hitherto, traditional methods of biomonitoring have been timely, but are limited by sampling and analysis methods. To overcome these limitations and achieve a more comprehensive surveillance biomonitoring at finely resolved spatial and temporal scales, molecular tools offer promising solutions. Our case study represents a proof-of-concept in transforming point-source eDNA into an informative ecological index that can be projected to the whole ecosystem scale (i.e. a river network) by the use of hydrological models. Importantly, this application could help align approaches not only within countries but also globally.

The ‘real-world’ application of eDNA and other molecular tools has grown considerably closer with concerted efforts from large consortia, and this is likely to improve greatly in the years to come [27,34,35,60,61]. Here, we discussed several potential avenues for molecular methods of biomonitoring and highlighted the importance of understanding both the constraints and the benefits of these methods, particularly in relation to macroinvertebrate biomonitoring. Of the traditional bioindicator groups typically used in freshwater, fish and diatoms are defined as single taxonomic groups, whereas macroinvertebrates represent several distinct taxonomic groups [30]. Therefore, approaches to target the DNA from this large ‘umbrella’ group must be suited for such high diversity (i.e. a multi-marker approach). However, as we have shown, even with a singular molecular marker it is possible to estimate an ecological index, and these tools provide further opportunities both to develop areas previously restricted by traditional methods (e.g. taxonomic resolution of overlooked taxa—Oligochaeta [40]) or to choose specific target key taxa within the macroinvertebrate group itself (e.g. Diptera, Ephemeroptera, Plecoptera and Trichoptera). Furthermore, macroinvertebrates encompass a plethora of not only diverse groups but also functions, which could be assessed via environmental RNA (eRNA). Environmental RNA, like eDNA, is indicative of the community richness, but unlike eDNA, eRNA is a contemporary signal and can be used to measure the expressed genes in a system (i.e. those required due to environmental conditions). Therefore, this upregulation in functional information from eRNA could be considered as an alternative to current species richness and measure the community response to environmental stressors at a functional level [62]. As eDNA is now frequently collected in aquatic systems to detect biodiversity in research, it is time to make the much-needed shift towards applying the eDNA-based assessment to ecological assessment [63]. This needs to include a concerted effort by researchers and end-users to not only intercalibrate eDNA with current traditional methods [27] but to go beyond both bioindicator groups [25] and simple point-based projections of ecological assessment, as shown in this study. The case study in this article represents an important step in the application of eDNA beyond point sampling and future research should incorporate sites across all classification classes (from bad to very good).

In conclusion, molecular methods, such as eDNA, will change the way in which we are able to examine biodiversity, its patterns and responses to environmental stressors at the ecosystem scale. Developing the translation of molecular data into ecological status is a vital part of harmonizing these methods into standard biomonitoring. Here, we illustrated a proof-of-concept in which we used hydrological models to project the point-source ecological information gained through eDNA and assess network-scale ecological status, which correlated significantly with traditional, point-based data on ecological status and river habitat information. This interdisciplinary approach to biomonitoring is reliant on expertise in several fields. We would therefore encourage cross-disciplinary work of ecologists, taxonomists, molecular biologists and hydrologists to fully exploit this new form of biological data. This form of collaborative working will not only boost the application of DNA-based biomonitoring, but also produces new concepts and approaches that can only aid our ability to conserve ecosystems from further degradation.

## Data Availability

All data used in this study can be accessed on the European Nucleotide Archive under project numbers PRJEB31920 and PRJEB33506. The analysis files used in this study are available at the following Github repository: https://github.com/lucarraro/eDITH_IBCH [64]. Supplementary material is available online [65].
